# Legislation for forensic investigative genetic genealogy in Sweden

**DOI:** 10.1016/j.fsisyn.2025.100637

**Published:** 2025-09-09

**Authors:** Ricky Ansell, Siri Aili Fagerholm

**Affiliations:** a Department of Physics, Chemistry & Biology (IFM), Linköping University, Linköping, Sweden; b The Swedish Police Authority, National Forensic Centre (NFC), Linköping, Sweden

**Keywords:** Forensic investigative genetic genealogy, FIGG, DNA, Law enforcement

## Abstract

Sweden was in 2019 the first country outside of North America to use forensic investigative genetic genealogy (FIGG) in solving a crime. However, further use of the method was inhibited in 2021 by the Swedish Authority for Privacy Protection Authority (IMY), following a mandatory prior consultation process. This decision was due to a considered lack of legislative support as well as issues regarding the transfer of sensitive personal data to a third country. Subsequently, method implementation was put on hold awaiting necessary legal review and amendments. In July 2024, the Swedish Justice Department presented a legal bill on the Police Authority use of FIGG, with only slight changes in relation to the considerations presented by a previous national committee on law enforcement use of biometrics. The legislation distinctly passed parliament vote February 2025 and it will gain force on July 1, 2025. Due to its sensitive nature the use of FIGG will be limited to the most severe offences; murder and aggravated rape, as a last resort and only following a prerequisite of “absolute necessity”. This paper describes the coming legislation on the use of FIGG in Sweden, discussing some parts of the constitutional comments and limitations seen, and describing implementation of FIGG within Swedish law enforcement.

## 1 Introduction

In 2018, law enforcement use of commercial DNA-based genealogy databases to solve crime as shown in the Golden State Killer case(s) [1],^1^ as well as to identify human remains took the public by surprise. This very useful method that has been publicly available for private users and professional genealogists to find relatives building pedigrees – why not find a perpetrator using the same approach as used to find a putative father?

The access and use of these databases is apparently a game changer from a law enforcement perspective providing the basis for an extremely powerful method – Forensic Investigative Genetic Genealogy (FIGG) – that helps acquire identity of unknown victims as well as suspects, and solve crime that would not have been solved by other investigative or forensic means.

**Fig. 1:**
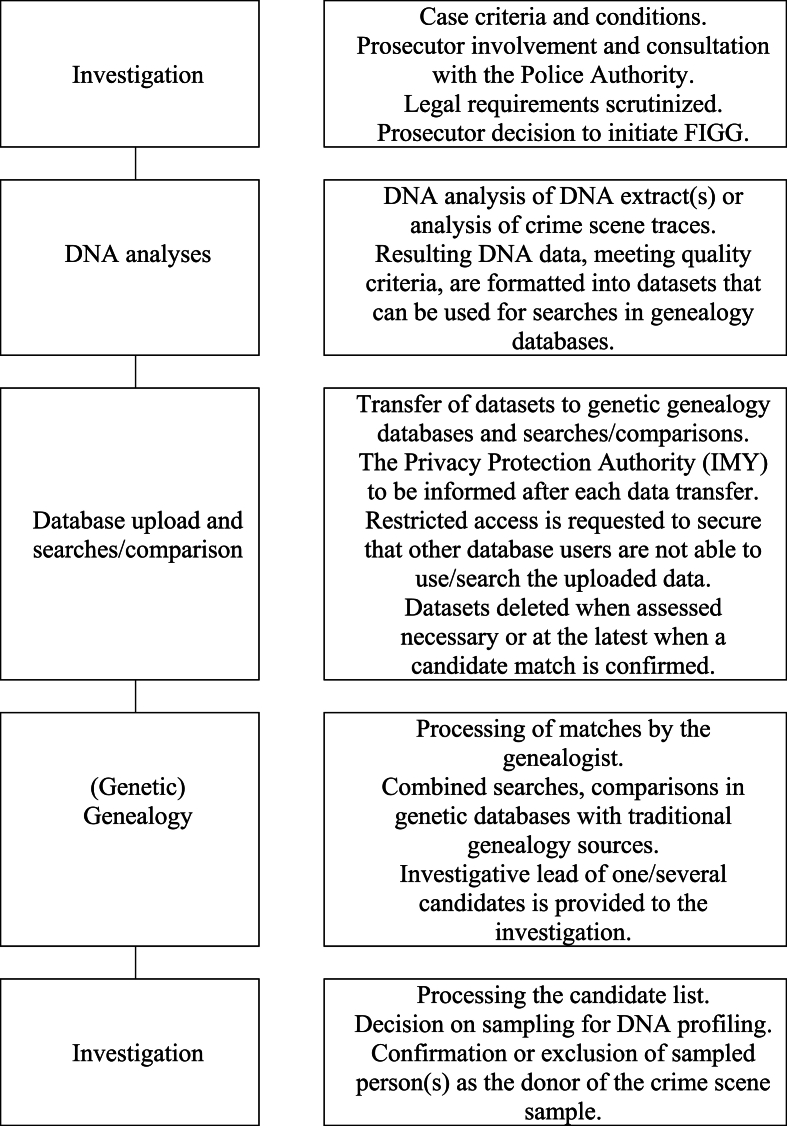
The different steps being part of FIGG according to the Swedish method with some of the details entailed. The person or persons of interest emerging from genealogy are to be reported as an investigative lead “*Forensiskt uppslag*” (forensic intelligence report), thus being regarded as intelligence information only, and not intended to be used as evidence. The final step includes verifying a match between the person(s) of interest and the crime stain DNA profile and is from a Swedish perspective considered an integral part of FIGG, as presented in the legal proceedings [40], thus expanding from the general view of FIGG e.g. [[2], [3], [4]].

Generalized, and as described in previous publications e.g. Refs. [[2], [3], [4]], the steps included in FIGG are; 1) generating DNA data (SNP profiles), 2) database upload and data comparison in commercial databases owned by private companies, followed by 3) genealogy (see Fig. 1). A major difference between consumer versus law enforcement use of the databases regards sample quality and quantity which affect the possibility and options to generate DNA data for the upload. This is generally not an issue for the private consumer who can provide a buccal swab or a spit sample to generate high quality DNA data. However, from a law enforcement perspective the quality and quantity of a sample can be challenging and may be a limiting factor. Common crime scene traces and samples collected from victims includes blood, hair, saliva, semen and contact traces. Many traces contain low amounts of DNA and may have been subjected to repeated STR (short tandem repeat) DNA profiling, resulting in low remaining volumes. It is also common with DNA mixtures, meaning that the DNA present originates from two or more individuals. This restricts usability of the samples. The quality of samples depends on for example the presence of analytical inhibitors, the degree of degradation (fragmentation) of the DNA and its proportion of single versus double stranded DNA. These factors may affect the analytical strategy when generating DNA data [[5], [6], [7]].

Two commercial genealogy databases, FamilyTreeDNA (FTDNA) and GEDmatch PRO, allow law enforcement access for FIGG purposes. Evidently, at the same time law enforcement started using these databases it provoked debate on legal grounds for its use, as well as ethical and privacy concerns and a need for policies and standardization. This is an ongoing debate which is valuable in increasing awareness and setting standards for the use of FIGG [e.g. Refs. 4,[8], [9], [10], [11], [12], [13], [14], [15]].

Primarily due to legal, ethical, privacy and practical (such as technological) concerns, alone or in combination, there have been an apparent and long lag-phase in implementing and using FIGG in countries outside of the U.S. FIGG has been used in criminal cases, as well as identification cases in both Australia and Canada, though still in moderate numbers [16,17]. Pilot cases have been run in Sweden, France, Norway, Estonia and Czech Republic [[18], [19], [20], [21], [22], [23]], are ongoing in the Netherlands and New Zealand [[24], [25], [26]], or are planned for as in the United Kingdom [27]. These pilot cases involve identification of deceased victims as well as cases to find a culprit through DNA left behind at the crime scene.

Outside of North America, Sweden was the first jurisdiction to use FIGG to try to identify human remains in a 2003 murder case (initiated in 2018) and to identify the culprit in an unsolved 2004 double murder case (in 2019) [19,28]. The double murder case became part of a formalized pilot case for which the scope was both to try to solve the case and also to gain a deeper insight in the different steps of FIGG, especially from a legal point of view [18,29].

The initial aim was to handle two unsolved cases to try to find the perpetrators, though only one case ended up being subjected to FIGG. The second crime was a severe rape case against an eight year old girl committed in 1995. This case was solved in 2019 just weeks before the FIGG pilot was activated, following new legislation allowing familial searches in the police DNA databases, “*Lag (2018:1693) om polisens behandling av personuppgifter inom brottsdatalagens område*” (Ch. 6, § 5) [30].

Familial searches can briefly be explained as an approach to use when a STR DNA profile of interest does not give a direct match in the Police Authority's DNA database [31]. The aim with the approach is to find close relatives through searches of the DNA profile in the Authority's own databases. Match candidates are presented as investigative leads to the preliminary investigation. The investigation must then examine whether any of the individuals on the list have a biological relative that can be of interest to the case. The approach is considered intrusive since none of the persons appearing on the list can be the donor of the DNA profile searched. In addition, close relatives may not be present in the database leading to examination of unrelated persons to the perpetrator. Most persons will only appear as a match candidate due to random similarities between the DNA profiles.^2^

A familial search performed in the Police registers in the rape case from 1995 led the investigation to the perpetrator. It turned out being the perpetrators son being registered in the convict DNA database. Even though familial searches are not allowed in many jurisdictions, the outcome of this case is of interest. If available, this strengthens the argument for using STR DNA profiles already obtained in the investigation with in-house analytical methods before turning to FIGG.

The remaining case, the 2004 double murder case, was successfully solved in 2020 using FIGG [18,19,29]. However, the identification case from 2003 has so far not been successful despite using FIGG and it remains unsolved [32].

In 2021, following a mandatory prior consultation process,^3^ the ongoing implementation of FIGG within the Swedish Police Authority was inhibited by the supervisory authority IMY (the Swedish Authority for Privacy Protection) [33]. The main issues addressed in their consultation statement were considered lack of legal support for the police to conduct searches in third-party databases and the processing of sensitive personal data, as well as concerns on the repeated transfer of sensitive (DNA) data to a “third country”^4^ and to private companies with regard to alleged insufficient data protection measures. Thus, in short it was concluded by IMY that legislative amendments were required in order for the Police Authority to gain legal authority to use FIGG. Obtaining this statement, the Police Authority refrained from using FIGG awaiting relevant legal amendments.

Legislations on law enforcement use of DNA-based genealogy databases or the handling of samples for such databases are still uncommon. The use of FIGG is regulated in a few states in the U.S. [13,34]. Denmark has so far not planned or run any FIGG pilot cases but is, as the second European country after Sweden, expected to have a legislation in place during 2025. Proposed provisions in the Danish legislation for FIGG is to be used to identify culprits in murder and attempted murder cases, aggravated rape and cases of terrorism^5^ [35].

In this paper we focus on describing and discussing the FIGG legislation gaining force in Sweden on July 1, 2025, by presenting the legislation in detail and in some parts examine the essence of the arguments put forward in the legal bill and the Biometric Committee Report. In brief, for a wider context the coming FIGG legislation will be described in relation to other legal amendments on law enforcement use of biometrics that are introduced in parallel.

### 1.1 The Biometric Committee and the steps towards legislation

A national committee on modernizing and harmonizing legislation for law enforcement use of different biometrics modalities was launched in Sweden in spring 2021 [36]. More specifically, the focus was to propose means for collecting and registering biometric data such as DNA, facial images and fingerprints in a uniform, comprehensive and appropriate way and in an expanded number of investigations (i.e. less severe criminal offences). In addition, the assignment included to provide proposals on the use of additional biometric modalities. The overall objective was to make it possible for more perpetrators to be identified and brought to justice.

**Fig. 2:**
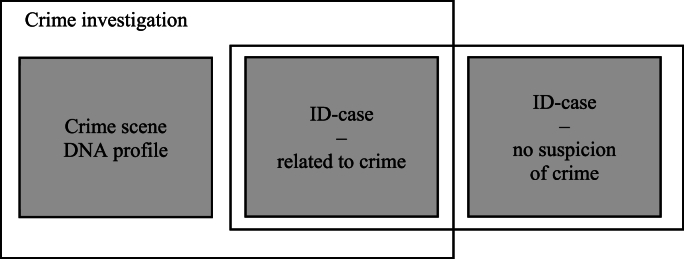
The Swedish legislation on FIGG does explicitly exclude identification of human remains – only permitting finding a perpetrator through DNA left behind at a crime scene. Samples from ID-cases related to a crime or with no suspicion of crime cannot be solved using FIGG, leaving a number of cases potentially unsolved and not using the full potential of the method.

As the implementation of FIGG was inhibited by IMY in May 2021, the Justice Department processed and added another assignment to the Biometric Committee in early 2022: to perform an overview on the use of FIGG and to consider and present legislative changes required for its use. Notably, the task given to the committee did not cover the identification of unidentified remains of crime nor identification of human remains (ID-cases) despite these areas having been clearly highlighted in the pilot case report as obvious and important applications of FIGG (Fig. 2) [18]. Thus, the task given was limited to making an overview of the present legal grounds and providing necessary provisions and amendments to use FIGG only to identify a perpetrator through DNA left behind at the crime scene. Repeated attempts by the Police Authority to include ID-cases in the legal overview were not successful.

The Biometric Committee presented their report in early June 2023 [36,37]. Based on the report, the Justice Department produced a draft legal bill that was presented in July 2024 [[38], [39], [40]] proposing major changes on biometric use by the police, and also including provisions on the use of FIGG in criminal investigations. The bill was proposed to gain force on July 1, 2025. Following common practice, the draft legal bill was sent for review at the Council of Legislation (*Lagrådet*) for consideration regarding consistency with the constitution and general legal principles. In September 2024 the Council stated they had no objections to the bill.

Subsequently, the legal bill was finalized [40] and parliamentary debate and vote took place at the end of February 2025 [41]. The legal bill passed with broad parliamentary support including support from all parties in opposition. One of the right-wing parties proposed a more extensive use of FIGG (to be applicable also for less serious crime cases). This proposal gained a number of votes.

Before final implementation of FIGG in Sweden, the process planned must be outlined in great detail and undergo a privacy risk assessment by the Police Authority legal advisors, followed by yet another prior consultation procedure involving IMY.

### 1.2 The amended biometric legislation

**Fig. 3:**
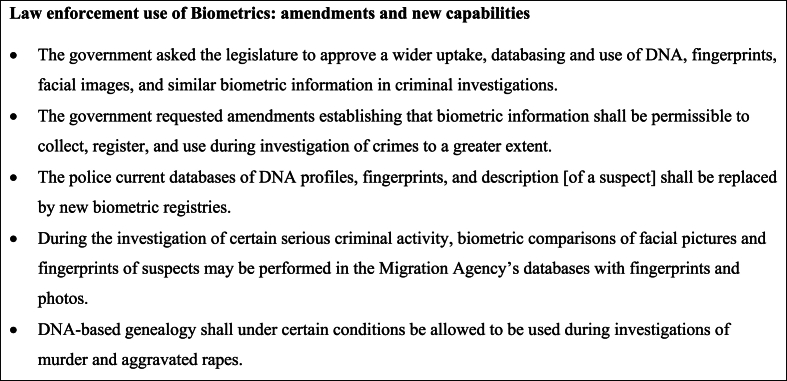
The scope and overall changes in the Swedish legislation on amended law enforcement use of biometrics as presented in the legal proceedings [40]. The government reasoned that DNA, fingerprints, facial images and similar biometrics that can be used for biometric analysis often are of crucial importance in identifying people who have committed crimes. Several of the changes implies infringement on the fundamental rights and freedoms of individuals. However, the infringements are necessary and proportionate to the objectives pursued and the government argues that there is a clear need to deal with the increase in gang-related crime and negative crime trends experienced, and that the increased opportunity that comes with technological development needs to be used as far as possible, without making disproportionate interventions in the protection of rights. Thus, in order to solve more crimes and bring more perpetrators to justice, law enforcement agencies need to be given increased opportunities to use biometrics in fighting crime. All proposals are argued necessary and justified based on that purpose. They are also argued as well balanced in relation to the intervention in the protection of rights that the proposals entail, and in addition the changes are also designed in such a way that they will protect individuals from arbitrary interventions.

For a brief summary of the government's aims, changes and reasoning behind the new expanded biometric legislation see Fig. 3. The legislation on using FIGG forms a small part of a larger package on amended law enforcement application of biometrics [40]. The aims of this legislative package include harmonizing in between present biometric modalities (DNA, fingerprints and facial images), but also to future-proof by opting for other biometric features (for example footprints, ear prints, handwriting, voices) some of which are not yet possible to implement without creating functional databases.

With the expanded law enforcement use of biometrics comes legal provisions also to use external (non-law enforcement) databases. Apart from DNA-based genealogy databases these include the Migration Agency (*Migrationsverket*) database that contains facial images and fingerprints. In a preliminary investigation concerning certain serious criminal offences, access to external databases may be authorized following a decision by the public prosecutor [40].

In the Biometric Committee Report an expansion allowing for law enforcement agencies to search the national passport register was suggested.^6^ This register is vast and covers approximately 6.3 million Swedish citizens (of a population of 10.5 million). If allowed to search and compare evidence facial images of a suspect (obtained from CCTV or other sources) with the registered images in the passport register, a match would, amongst other information provide name and address [36]. This would provide law enforcement agencies with potentially highly important information, and from a database with vast population coverage. This suggested change provoked some immediate reactions amongst referral bodies and media. Turning the passport register into a biometric database searchable by the Police Authority was for example considered by IMY to be excessive and intrusive [42], and this expansion did not become part of the legal bill.

### 1.3 The FIGG legislation

**Fig. 4:**
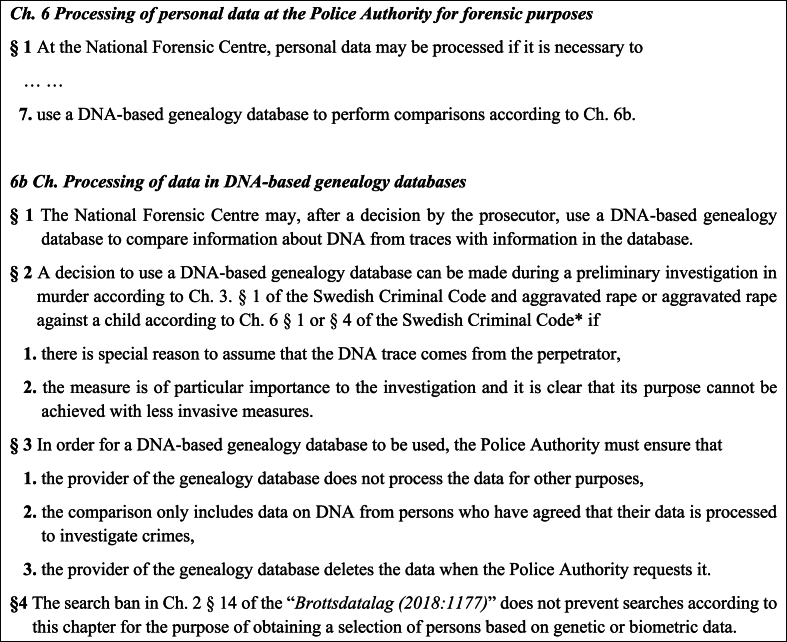
A translated excerpt of the legislation related to the use of FIGG in Sweden: *“Lag (2018:1693) om polisens behandling av personuppgifter inom brottsdatalagens område”* [30]*.* The requirements set out in the third paragraph apply in addition to what otherwise follows from the data protection regulation (“*Brottsdatalag (2018:1177)*” [43], including the requirements on the obligations of the person responsible for handling of personal data, in this case genetic data (Ch. 3.), and on the transfer of personal data to a third country (Ch. 8.). ∗ “*Brottsbalk (1962:700)*”.

The Swedish FIGG legislation is detailed and strict but at the same time clear and straightforward (Fig. 4). It is supported by background proceedings describing its use in further detail [40]. The context of the legislation can be clearly condensed as a single paragraph, as follows: the legislation “*Lag (2018:1693) om polisens behandling av personuppgifter inom brottsdatalagens område*” is provided with a specific provision (Ch. 6 b). With the amendment made, the National Forensic Centre (NFC), a division of the Swedish Police Authority, has the legal support to use DNA-based genealogy databases [30]. This provision lifts a general data protection search ban for the purpose of obtaining a selection of persons based on genetic or biometric data using such databases. NFC may, after a decision by a prosecutor, use a DNA-based genealogy database to compare information in the database with DNA data from traces. Such a decision can be taken during a preliminary investigation of murder or aggravated rape (adults or children),^7^ if there is special reason to assume that the DNA trace comes from the perpetrator, for example semen found at the crime scene of a sexual assault or blood on a knife not matching the murder victim. Further, the measure must be of particular importance i.e. “absolute necessity” to the investigation and predicted that the same outcome cannot be achieved with less invasive means. When using these databases, the Police Authority must ensure that the provider of the database does not process the data for any other purpose than intended, that the comparisons are made only with persons who have opted in, i.e. agreed that their data is processed in order to investigate crimes, and that the database provider deletes the data entered upon request.

In addition to the paragraphs directly mentioning FIGG, additional legislation “*Brottsdatalag (2018:1177)*” applies that covers the obligations of the person in charge of personal data (Ch. 3) and the requirements on the transfer of personal data to a third country (Ch. 8, the same law) [43].

### 1.4 Should police-led genealogy be considered investigative or forensic (or both)?

One major question raised already in the legal inquiry made by the Police Authority preceding the pilot case was on the data handled in the genealogy databases and the steps performed by a genealogist and in particular whether these actions constitute processing of genetic data (forensic activity) or could be considered processing of personal data (investigative activity) [44]. This was of significant importance for the lawmakers as to where in the legislation the provisions should be positioned.

The existing legislation directs exclusive responsibility for the police processing of genetic data to the NFC [30]. Thus, NFC constitutes the sole entity within the Police Authority to handle such (sensitive) information. As a consequence, the head of NFC holds the ultimate responsibility for the processing of genetic data in the databases (Ch. 6b. §1).

The Biometric Committee Report discusses the handling performed by the genealogist and concludes the use of the genealogy databases involves (repeated) searches and handling of information and, from a Swedish perspective, this information contains DNA data meaning that this part of the process is to be considered as a forensic activity and not an investigative activity. As a consequence, the legal provisions on the use of genetic genealogy databases are positioned in the forensic chapter of the legal act.

For one thing, this means in practice that a genealogist recruited or hired by the Police Authority would need to perform their work under the auspice of the NFC. Another consequence is that since the responsibility for processing genetic information lays on the head of the NFC, any decision on using FIGG cannot be taken by the investigation leader (prosecutor) without obtaining clearance from the NFC. This relates to questions on correct handling of genetic information for which the NFC must guarantee that suitable conditions for data processing prevail, including that the prerequisites for third country transfers are met as stated in “*Brottsdatalag (2018:1177)”* (Ch. 8) [43], and discussed in more detail below.

In short, from a Swedish perspective, this would also rule out the use of the acronym IGG in favor for FGG. However, considering the way this method is described in preceding works as well as the Swedish legal bill, the use of IFGG or FIGG would appear more appropriate. This is because the process involves measures of an investigative nature, not merely forensic. Given the legal framework, IFGG would appear to be the appropriate term to use to describe the method, as discussed earlier in Tuazon et al., 2024 [45].

Despite being considered a forensic task from a legal perspective, it is obvious that interactions between the genealogist and the investigation team are needed as the work progresses in order to discuss findings when building the pedigree to bring the case forward.

### 1.5 Investigative and forensic measures to be taken

For FIGG to be used it must not only be of particular importance to the investigation, but it must also be established that the objective cannot be achieved with less intrusive measures. In addition, the lawmakers stress that extensive investigative and forensic measures must have been performed beforehand in the investigation [40].

Amongst forensic measures being exemplified by the lawmakers are national and international (Prüm) database searches, familial searches in the national DNA database, extended DNA screens and analyses regarding biogeographical ancestry and physical appearance (eye-, hair- and skin color) [40]. The possibility conducting these measures or obtaining useful results is not always straightforward.

Performing database searches, including familial searches, should be an obvious measure, as a STR DNA profile is typically available and the necessary tools readily accessible. Though as discussed previously, in some jurisdictions familial searches are not permitted as is the case in Norway [21].

Performing an extended DNA screen, also called a DNA mass screen, can be perceived as intrusive and may not be suitable or practically possible in all investigations.

Regarding attempts to predict ancestry and physical appearance from DNA, such predictions could provide important information. Such information is crucial for evaluating the applicability of FIGG, as it provides insight in the specific case in relation to the population representation within the genealogy databases, as well as the availability and reliability of civil and genealogical records from the region of interest, both of which are essential for accurate genealogical reconstruction. Testimonies from witnesses or information from other sources on the perpetrator's possible ancestry should be considered with caution if the information exist. From the lawmaker's perspective these databases are not expected to be used unless the investigation can conclude that the use will bring the case forward. Obviously, this can be regarded as a complicated limitation. In addition, a relevant population does not by itself guarantee that there will be close enough relatives in the database to perform fruitful genealogy research.

In addition, and as earlier described, sample quality and DNA quantity may be limited, not allowing all available analyses to be performed.

### 1.6 The use of FIGG must be decided by a prosecutor

One major difference between the amended legislation and what was initially suggested by the Biometric Committee regard who should decide whether or not to use FIGG in an investigation. The legislation clearly states that a prosecutor must make this decision [40]. In the Committee Report this was not transparently stated, which implies that according to the Committee this decision may just as well be taken by a police investigator - if the preliminary investigation in question was police-led [36]. In Sweden, police-led preliminary investigations are common practice in cases without a suspect as typically seen in cold case murder investigations (in which a prosecutor may be involved more as an informed bystander ready to intervene if necessary).

In the legislation it is explicitly stated that a prosecutor must make the decision on using FIGG (Fig. 4). This can be interpreted as the lawmakers seeking to restrict the use of FIGG. It can also be seen as a compromise on behalf of the lawmakers, from the perspective that this approach also takes into account suggestions by IMY and other referral bodies (such as the Swedish National Courts Administration) that a mandatory court review be required before any decision to use FIGG. The main argument for including a court review in initiating a FIGG case was to ensure that all legal prerequisites were met in each individual case. In the case of IMY, this solution was argued for previously in a supplementary special statement in the Biometric Committee Report (given by the IMY representative) [36]. The government argued in the legal bill [40] that despite the method being privacy-sensitive, a system involving a court review appears neither suitable nor expedient. They argued that the method involves traces (DNA from an unknown suspect) for comparison (to identify the unknown suspect) and can therefore not be compared with covert coercive measures against an individual (known) in which a court review typically can be justified. Another important referral body, the Swedish Commission on Security and Integrity Protection (SIN)^8^ suggested the solution that a prosecutor should make the decision. The solution chosen by the lawmakers can be seen as a pragmatic compromise that addressed the concerns raised by IMY and others.

Therefore, a preliminary investigation on murder or aggravated rape that is not led by a prosecutor will need to enroll one in order to decide on using FIGG.

In addition to this, the prosecutor's role in a FIGG case will not be as independent as it is in a general preliminary investigation. This is because a decision to use FIGG can only be taken following a mandatory consultation with the Police Authority. This is, as described in the legal bill, because the Police Authority is the party responsible for assuring that legal requirements to use FIGG are met in each individual case. The consultation procedure can be seen as a variant of the advisory board that was suggested previously in the pilot case report [18]. This group within the Police Authority is expected to include high ranked officers with representation from the National Operations Department (NOA) and the NFC. The group is expected to be supported by a reference or expert group with different competencies such as investigators, DNA and legal experts, as well as a genealogist. The Police Authority will also be responsible for any prioritization between different possible FIGG cases if any necessary resource becomes a limiting factor. Altogether, the decision making in activating a FIGG case is elevated and surrounded with protective measures.

If this task will be appointed to a prosecutor handling murder or rape cases in general, or if it will be appointed to dedicated “FIGG prosecutors” is not yet decided. It also remains to be seen if a prosecutor appointed to a FIGG case will stay in charge for the continuation of the preliminary investigation, or if the enrollment is to be temporary and end after the decision to use FIGG, or if this will depend on the case. Once the decision to use FIGG has been taken by the prosecutor, a request will be sent to NFC and the work can begin.

### 1.7 Transfer of DNA data to a “third country”

Transfer of personal data to a non-EU member state – a third country – is regulated by EU law. The basic rule is that such transfers are to be directed to a competent (mandated) authority^9^ in the recipient country [44,46]. With key genealogy databases FTDNA and GEDmatch PRO located in the U.S. a competent authority would be the FBI.

The FIGG process developed in Sweden does not involve the use of a competent authority in the recipient country. Thus, from a Swedish perspective, a prerequisite for law enforcement use is that there are other legal provisions in order to transfer the DNA data to a private actor without involving a competent authority. The question on the need to transfer personal data to a third country, was indeed one of the main concerns addressed by IMY and later also discussed by the Biometric Committee [33,36].

The position presented in the legal bill on third country DNA data transfer to use FIGG [40], can be briefly summarized as follows: the main rule according to the Law Enforcement Directive (LED) is that a transfer of personal data to a third country must be made to a competent authority (Article 35), but a EU member state has the right to stipulate that such data, in individual and special cases, may be transferred directly to recipients in a third country (Article 39) [46].

In Sweden, the LED's provisions on third country transfers are implemented in “*Brottsdatalag (2018:1177)*” (Ch. 8) [43]. One provision constitutes an exception from the general requirement that third country transfers must be made to a competent authority, thus providing the option to transfer personal data directly to another recipient in a third country, but only in individual cases where specific criteria are met (Ch. 8, § 8). In addition, personal data can only be transferred if other general prerequisites in the Criminal Data Act are fulfilled (Ch. 8, § 1 and § 2). Another requirement is that the transfer must be *absolutely necessary* for the Police Authority to be able to perform the tasks for which it is responsible (Ch. 8, § 8.1), in this case to prevent or detect criminal activity, investigate or prosecute crimes or enforce criminal penalties, or to maintain public order and safety (Ch. 1, § 2). It is also required that the authority inform the recipient (the database suppliers) on the specific purpose for which the data may be processed (Ch. 8, § 8.2), and that it would be deemed inefficient and/or inappropriate to transfer the data to a competent authority in the third country (Ch 8, § 8.3). In the legal bill it is on these bases concluded that: “*The provisions in LED on third country transfers therefore do not constitute an obstacle to the introduction of provisions that allow investigative genetic genealogy to be used*” [40].

It will be the transferring authority, in this case the Police Authority, that will decide in each individual case whether the legal conditions for a transfer of DNA data directly to the provider of the genealogy database are met including that requirements regarding “absolute necessity” are fulfilled (this being part of the consultation process the prosecutor is obliged to have with the Police Authority before a decision to use FIGG).

With the design of the FIGG regulation, the government assesses for its part that the requirement that the transfer must be absolutely necessary should normally be considered fulfilled if and when a decision has been made by the prosecutor that FIGG is to be used in an investigation. This is because in such a case, an assessment has already been made that there are no other measures of less intrusiveness and intervention that can be used to bring the criminal investigation further towards resolution.

The same applies to the requirement stated in the legislation that personal data may not be transferred if the data subject's interest in protection against violation of their rights and freedoms outweighs the public interest in the transfer taking place (Ch. 8, § 8) [43]. Therefore, it is concluded that FIGG shall only be used in investigations of particularly serious crimes where the public interest in effective suspect identification outweighs the breach of privacy that the measure entails for the individual. In this perspective, crimes that are considered the most grave are murders and aggravated rape of a child (no prescription time and high public interest to solve), and aggravated rape (crimes which can lead to long sentences and being of high public interest).

When examining the requirement that it would be inefficient or inappropriate to transfer the personal data to a competent authority, it should be taken into account, among other things, whether such a transfer can take place with the same security for the personal data and efficiency as if these were to be transferred directly to the database. In the legal inquiry preceding the Swedish pilot case the Police Authority argued that these circumstances did indeed apply [44] and this point of view has remained the same [36,38,40]. In addition, measures to secure proper treatment of personal data in the genealogy databases must be taken by the Police Authority (Fig. 4).

For each data transfer, NFC is obliged to notify IMY (being the supervisory authority) in writing on any transfer made to a third country (Ch. 7, § 5, “*Brottsdataförordningen (2018:1202)*”) [47]. The information given must include: 1) date of transfer, 2) receiving party, 3) reason for transfer and 4) kind of data/information transferred. In effect, this notification will give IMY the means needed to review the use of FIGG and to control data security measures taken by the Police Authority.

### 1.8 Comparative searches in the genealogy databases

The legal inquiry by the Police Authority preceding the pilot case also addressed comparative searches in genealogy databases. Article 10(c)^10^ in LED [46] was referred to as the grounds for the searches in the pilot case. Though at the same time the legal adviser argued that the legal grounds needed to be investigated further [44]. Subsequently, IMY in their consultation statement concluded a lack of legal support for the searches [33]. However, the Biometric Committee found the legal grounds for this part of the method difficult to assess and left the question open for legal review. In the legal bill, the Justice Department argued that genetic and biometric data can be handled by the NFC but the current law lacked the specific purpose. Thus, in the legislation such specific purpose has now been introduced (Ch. 6, 1§ 7) “*At the National Forensic Centre, personal data may be processed if it is necessary to … use a DNA-based genealogy database to perform comparisons according to Ch. 6b*”. Chapter 6b is a new subchapter specific for FIGG describing the prerequisites (see Fig. 4) [30].

In a recent paper by Kuru (2024) [48], focusing on FIGG in the European (EU) perspective it is concluded that the legal basis in the EU data protection framework, specifically Article 10(c) of LED, does not support the use of FIGG. In brief, the legal basis “*manifestly made public by the data subject*” cannot be relied on for accessing personal data of genetic genealogy database users for investigative purposes. This is because law enforcement use in relation to the information given fails to meet the requirements. Further, it is argued that a proper union or member state (i.e. national) law following Article 10(a) in LED would be the best option to provide legal support for FIGG, which is in line with the Swedish legal amendments made.

### 1.9 Potential implications regarding minors

In a recently published Swedish Committee Report on legislative updates regarding coercive measures on minors, entitled ‘*More effective tools to counter crime by young offenders*’, it was discussed whether the use of FIGG both in terms of minor culprits and minors being registered in the genealogy databases was consistent with law [49]. This topic was not explicitly discussed and considered in the Biometric Committee Report [36]. Amongst matters considered was the fact that members of the public signing up for consumer DNA-based genealogy databases self-state their age, so their actual age cannot be controlled or guaranteed. Obviously, the assessment made by the Committee in the young offender report could have had a negative impact on the utility of the Swedish FIGG legislation, or even inhibited the use of FIGG.

It was concluded that the common interest of an effective preliminary investigation of the most severe crimes (as those covered by the FIGG legislation) weighs heavier than a child's need for personal integrity. The basis for this assessment is referred to in the report [49]: first, it is considered that if minors were to be excluded from the databases or searches it would have counteracted the results aimed for in the proposed legal bill on the use of FIGG. Second, a legal guardian needs to consent for their child's sample to be entered on the databases and used for matching (opted in) by law enforcement, and third: the method will only be used in a few selected cases of serious crimes and when absolutely necessary for the investigation. Combined, the treatment is not considered to be in conflict with the European Convention on Human Rights and the Conventions on the rights of a child, meaning that no exceptions for minors were considered needed.

## 2 Summary

More than six years after the first use of FIGG in Sweden, legal grounds is introduced that explicitly permit its use in a selected number of murder investigations and aggravated rape cases under strict requirements [40]. Arguments on proportionality versus the sensitive nature of the method limits the use to only the most severe crimes. Clearly the lawmakers see FIGG as an intrusive and extraordinary method to be used with restriction and as a last resort. The principle of proportionality should permeate the work of law enforcement agencies. However, it is clear that what constitutes a proportionate response is inherently subjective.

Overall, the introduced legal paragraphs covering FIGG are detailed and quite specific in guiding law enforcement to use the method (Fig. 4). Not only is the method restricted to the most serious crimes, it is also expected to be used in a limited number of cases and only after other investigative and forensic measures have not been successful and it is clear the case cannot be advanced or solved with less invasive measures.

FIGG as a method has been described previously e.g. [[2], [3], [4], [21]], and given the legal proceeding [40] the Swedish view of FIGG is broader than previous descriptions in other jurisdictions. In Sweden the method comprises all measures from the initial process of reaching a decision to use FIGG to the final investigative efforts made that includes verifying the match between the person of interest and the crime scene DNA profile (Fig. 1).

In the decision process the prosecutor is obliged to have a consultation with the Police Authority. This alternative seems reasonable and avoid the delays likely to result should court hearings be required. With a mandatory consultation process involving the prosecutor and the Police Authority, it will be possible to prioritize between cases (which is the responsibility of the Police Authority) and thereby achieve a uniform treatment nationwide, but also to obtain an overview of cases on a national basis.

The lawmaker's view of FIGG being intrusive and a last resort is also apparent when mentioning forensic measures to have been taken before considering to use FIGG [40]. Some of these measures are easier and more straightforward to implement than others. As an example, existing STR DNA profiles can be used for national and international (Prüm) database searches and familial searches. These measures will not consume any of the DNA sample. Whereas separate analyses for biogeographical ancestry and physical appearance obviously will consume DNA and potentially not result in a useful outcome. The requirement that genealogy databases only can be used if the population of interest is part of the database is inexplicit and blunt. These limiting factors may conflict with the practical realities of casework, thereby restricting the applicability of FIGG. It remains to be seen how this will be handled in practice.

Regarding the crime categories defined in the legislation [40,43], this is an obvious limitation compared to the crime categories possible for law enforcement as stated in the guidelines and user policies of the genealogy databases FTDNA and GEDmatch PRO that both allow searches in a broader range of violent crimes.^11^ As previously discussed, and argued for by the lawmakers, only the most severe crimes will meet the requirements for proportionality on the processing of sensitive personal data as done using FIGG. Obviously, the lawmakers with this in mind did not consider FIGG in perspectives other than being a last resort based on case-by-case specific needs. Thus, it will for example not be possible to use the method in an early investigation phase, not even with urgent needs that might be apparent in serial murder or sequential rape cases.

In the legal bill, the data protection requirements for the processing of sensitive personal data are discussed in relation to how much DNA data to transfer to genealogy databases [40]. According to the legislation, the amount of sensitive personal data to be processed must be limited and based on the purpose of the processing (Ch. 2, § 8) [43]. For FIGG, this means that the amount of data that is transferred must be limited in such a way that it does not contain more data than is necessary to enable the search for relatives in the database. Obviously, that amount of data may vary and will not be known beforehand. Repeated transfers (and subsequent data deletions) with an increasing amount of data being uploaded is considered sensitive and therefore not seen as a sensible alternative. Without knowing the search outcome beforehand, the solution will simply be to transfer the data set available. From the same perspective, but not explicitly discussed in the legal bill, is the timeframe allowed for the processing of data in the databases, i.e. keeping data uploaded. How long would a sensible processing time be until the data should be erased? Obviously, some FIGG cases will be successful within a narrow timeframe whereas others will not succeed at all and could in theory go on for years. Such circumstances would need to be considered from this point of view when shaping a strategy on when to close a case (i.e. delete data from the database).

## 3 Considerations and future perspectives

Overall, the success of using DNA-based genealogy databases in ID-cases outnumbers crime scene cases [50]. The pilot case report proposed the application of FIGG to ID-cases, given its proven ability to assist in identifying unknown persons [18,29]. Unfortunately, and presumably with a large impact, the identification of unknown human remains (with or without connection to a crime) will not be allowed in Sweden. In the legal bill it is explicitly stated that: “*The provisions do not allow a DNA-based genealogy database to be used for the purpose of, for example, identifying a deceased victim*” [40]. For unknown reasons ID-cases were never introduced by the Justice department in the Biometric Committee directives. In light of the focus of the Committee's directives on increased identification of perpetrators with biometrics, this added task may have been considered somewhat out of scope.

Despite this, it can be argued that using FIGG to attain an ID of a deceased person is less intrusive than finding a culprit through their DNA left behind at the crime scene. Apart from aiding the criminal investigation by naming an unknown and what that may bring to the case, other important values are added such as family members being notified of their missed relatives and to be able to put a name on a grave. It remains to be seen, but it is expected that the Swedish Police Authority will assess the legal grounds for this application and request a legal review and necessary amendments by the Justice Department. The legal basis as a starting point is expected to be GDPR (the EU General Data Protection Regulation) rather than LED.

In the Swedish pilot case report it was stated that normative national guidelines would be one way forward to achieve a standardized and at the same time ethical, sound and reasonable use of FIGG [18]. With legislation that in much detail directs its use, one could argue that the same need for a normative guideline is no longer present since most essential parts are already covered and clearly expressed. Remaining parts could instead be covered by an informative guideline or standard operating procedure describing the FIGG method. The NFC and NOA would be responsible to prepare and oversee such documents.

As an increasing number of European countries are starting pilot cases or are proceeding towards implementation of FIGG [[20], [21], [22], [23], [24],^27^], it will be interesting to follow any changes or amendments in national legislation and to what extent variants in different legislation appear. Within the European Union the legal framework is the same, but on a national level variants in legislation are still possible. This powerful yet intrusive method, is expected to be implemented in an increasing number of countries following necessary legislative updates. To be used in certain cases to finally solve serious crimes and identify missing people, for justice and to set the mind at some rest for those who suffer.

## 4 Concluding remarks

The Swedish legislation on using FIGG is the first of its kind outside of the U.S. and it regulates to a detailed level the use of the method. It is clear that the lawmakers regard FIGG as highly intrusive with regard to privacy matters and therefore restrict the use to a limited number of murder and aggravated rape cases under the obligation of absolute necessity for the preliminary investigation. At this stage legislation does not include identification cases.

## CRediT authorship contribution statement

**Ricky Ansell:** Writing – review & editing, Writing – original draft, Project administration, Investigation, Conceptualization. **Siri Aili Fagerholm:** Writing – review & editing, Writing – original draft, Investigation, Conceptualization.

## Declaration of competing interest

The authors are active in the implementation of FIGG within the Swedish Police Authority, and declare that they have no competing financial interests or known personal relationships that could have influenced the work reported in this paper.

## Footnotes

1 By using a commercial genealogy database U.S. authorities found the so-called Golden State Killer - a man responsible for numerous rapes, murders, and countless home invasions that occurred in California during the 1970's and 1980's.

2 A familial search can be achieved by adjusting the search parameters in the DNA database admitting a certain level of mismatches. By doing so close relatives such as parent-child or full siblings can be found. The tool is blunt as it will generate irrelevant matches with unrelated persons registered in the database. Also, a full sibling might not appear as a top candidate of a search when candidates are ranked according to the likelihood of such a relationship. A full sibling might not even appear at all in the list despite the STR DNA profile being registered due to large differences between the DNA profiles.

3 Request for prior consultation (“*Förhandssamråd*”): Before each new type of personal data processing or significant changes in ongoing processing, law enforcement authorities must carry out an impact assessment if the processing can be assumed to entail a particular risk of infringement of personal integrity. A prior consultation with the Swedish Authority for Privacy Protection – IMY (*Integritetsskyddsmyndigheten*), is mandatory when the impact assessment shows that there is a particular risk of infringement of the data subject's personal integrity and/or the type of treatment involves a particular risk of infringement. Request for prior consultation is assumed to be made in good time before the processing begins or significant changes are implemented. https://www.imy.se/verksamhet/utfora-arenden/begar-forhandssamrad-enligt-brottsdatalagen/.

4 In this perspective a “third country” is a state not being a member state of the European Union or EEA (the European Economic Area).

5 The authors acknowledge that definitions for serious/aggravated rape as well as for terrorism vary between different jurisdictions.

6 According to present regulatory law, “*Passförordning (1979:664)*”, the Swedish Police Authority shall release a photograph of an individual from the passport register following a request from e.g. the Security Service (*Säkerhetspolisen*), the Economic Crime Authority (*Ekobrottsmyndigheten*), a Swedish embassy, the Armed Forces (*Försvarsmakten*), the Coast Guard (*Kustbevakningen*), the Customs (*Tullverket*), the Tax Agency (*Skatteverket*) and the Enforcement Authority (*Kronofogdemyndigheten*). https://www.riksdagen.se/sv/dokument-och-lagar/dokument/svensk-forfattningssamling/passforordning-1979664_sfs-1979-664/.

7 Aggravated rape is defined as follows in the Swedish Criminal Code (“*Brottsbalk (1962:700)*”) regarding adult victims: “*If a crime referred to in the first paragraph is serious, a sentence of imprisonment for aggravated rape shall be imposed for a minimum of five and a maximum of ten years. When assessing whether the crime is serious, special consideration shall be given to whether the perpetrator has used violence or threats of a particularly serious nature or whether more than one person has assaulted the victim or otherwise participated in the assault or whether the perpetrator, with regard to the manner of the act or the young age of the victim or otherwise, has shown particular recklessness or cruelty. Act (2022:1043).”,* and children: “*If an offence referred to in the first or second paragraph is serious, a sentence of imprisonment for aggravated rape of a child shall be imposed for a minimum of five and a maximum of ten years. When assessing whether the offence is serious, particular consideration shall be given to whether the perpetrator has used violence or threatened to commit a criminal act or whether more than one person has assaulted the child or otherwise participated in the assault or whether the perpetrator, with regard to the manner of the act or the child's young age or otherwise, has shown particular recklessness or cruelty. Act (2022:1043)*”. https://www.riksdagen.se/sv/dokument-och-lagar/dokument/svensk-forfattningssamling/brottsbalk-1962700_sfs-1962-700/#K6.

8 The Swedish Commission on Security and Integrity Protection (*Säkerhets-och integritetsskyddsnämnden*) supervises the processing of personal data by the Swedish Police, and that this is conducted in accordance with laws and other regulations. https://www.sakint.se/the-swedish-commission-on-security-and-integrity-protection/.

9 A competent (mandated) authority in a third country is an authority or organization in a country outside the EU/EEA (European Economic Area) that has authority to monitor and enforce data protection rules.

10 Article 10 of the European Union Law Enforcement Directive, Directive (EU) 2016/680 [46]: *“Processing of special categories of personal data Processing of personal data revealing racial or ethnic origin, political opinions, religious or philosophical beliefs, or trade union membership, and the processing of genetic data, biometric data for the purpose of uniquely identifying a natural person, data concerning health or data concerning a natural person's sex life or sexual orientation shall be allowed only where strictly necessary, subject to appropriate safeguards for the rights and freedoms of the data subject, and only:* *(a) where authorised by Union or Member State law;* *(b) to protect the vital interests of the data subject or of another natural person; or* *(c) where such processing relates to data which are manifestly made public by the data subject.”*

11 It is allowed for law enforcement purposes to use the services provided by FTDNA: to identify a perpetrator of a violent crime which means any “homicide (including murder or non-negligent manslaughter), any sex crime (including rape), or any of the following serious crimes or criminal offences: (1) armed robbery, (2) aggravated assault, or (3) any other violent crimes as defined by the FBI Uniform Crime Reporting (UCR) Program.” Attempts to commit such crimes are included. https://www.familytreedna.com/legal/law-enforcement-guide GEDmatch PRO: to identify a perpetrator of a violent crime “defined as murder, nonnegligent manslaughter, aggravated rape, robbery, or aggravated assault”. https://pro.gedmatch.com/terms-use-oct-2024.
